# ASO Author Reflections: Adjuvant Chemoradiation in Resected Biliary Cancers—Insights and Implications

**DOI:** 10.1245/s10434-024-15172-5

**Published:** 2024-03-12

**Authors:** Dana A. Dominguez, Laleh G. Melstrom

**Affiliations:** Department of Surgical Oncology, City of Hope National Medical Center, Duarte, CA USA

## Past

Adjuvant chemoradiation (CRT) is an option within the standard of care for patients with extrahepatic biliary cancer, including gallbladder cancer after resection. However, strong evidence supporting its use remains limited, with the most notable support coming from the SWOG S0809 trial, a single-arm study of 79 patients demonstrating improved overall survival using gemcitabine/capecitabine followed by capecitabine CRT compared with historical survival rates.^1^ Adequately powered randomized clinical trials in these populations are a significant challenge due to the relative rarity of these histologies, often requiring the combining of disease sites, which creates trial heterogeneity, making comparative interpretation of evidence more difficult.

## Present

The current study published in this issue of the *Annals of Surgical Oncology* aimed to provide further corroboration of the SWOG S0809 trial using the National Cancer Database (NCDB) with strict selection criteria to reproduce the study conditions. In a cohort of 469 patients with resected extrahepatic cholangiocarcinoma (gallbladder cancer), the authors demonstrated a similar median overall survival of 36.9 months for patients receiving adjuvant CRT.^2^ Additionally, the study highlights important prognostic factors in patients treated with CRT, demonstrating that both positive nodal status and resection margin were associated with abbreviated overall survival. Finally, in an entropy-balanced cohort comparing adjuvant chemotherapy alone with CRT, CRT demonstrated improved overall survival. Although limited by constraints of a retrospective database, most notably chemotherapy regimen specifics other than the number of agents used, the study’s results were strikingly similar to those found in the SWOG S0809 trial, providing further evidence to support the continued use of adjuvant CRT in clinical practice, especially for higher-risk patients.

## Future

Future research must focus on prospective trials to validate the benefits of adjuvant CRT in biliary cancers. Some further information may come from ACTICCA-1, a phase 3 randomized clinical trial evaluating adjuvant chemotherapy with gemcitabine and cisplatin compared with capecitabine, which in 2021 addended its protocol to include an embedded cohort of patients with R1 resections randomized to either chemotherapy alone or CRT.^3^ Investigating the molecular and genetic characteristics of biliary cancers also could provide insights into tailored therapeutic approaches and improve patient stratification for adjuvant therapy, especially with immunotherapy already incorporated into the management of unresectable/metastatic biliary tract cancers with the more recent TOPAZ-1 trial.^4^
